# Hidden in plain sight: two co-occurring cryptic species of *Supplanaxis* in the Caribbean (Cerithioidea, Planaxidae)

**DOI:** 10.3897/zookeys.991.57521

**Published:** 2020-11-11

**Authors:** Ellen E. Strong, Philippe Bouchet

**Affiliations:** 1 National Museum of Natural History, Smithsonian Institution, PO Box 37012, MRC 163, Washington, DC 20013-7012, USA Smithsonian Institution Washington United States of America; 2 Institut de Systématique, Évolution, Biodiversité, ISYEB, UMR7205 (CNRS, EPHE, MNHN, UPMC), Muséum National d’Histoire Naturelle, Sorbonne Universités, 43 Rue Cuvier, 75231 Paris Cedex 05, France Sorbonne Universités Paris France

**Keywords:** distribution, radular morphology, shell morphology, synonymy

## Abstract

The cerithioid *Supplanaxisnucleus* (Bruguière, 1789) is widespread in the Caribbean, where it lives in often dense aggregates on hard surfaces in the middle-high intertidal. Molecular evidence shows that it comprises two species that are in fact morphologically diagnosable. We fix the nomenclature of *Supplanaxisnucleus* by designating a sequenced neotype from Bruguière’s historical locality of Barbados, and identify the second, cryptic species as *S.nancyae* (Petuch, 2013). The two live syntopically across the Caribbean and form a closely related species group with the Panamic *S.planicostatus* (G.B. Sowerby I, 1825). *Planaxisnucleola* Mörch, 1876, described from St Croix, in the Virgin Islands, never again recorded in the literature but listed as a synonym of *S.nucleus* in taxonomic authority lists, is recognized as a valid species of *Hinea* Gray, 1847. *Proplanaxis* Thiele, 1929 and *Supplanaxis* Thiele, 1929, are synonyms and the latter is given precedence over the former.

## Introduction

*Supplanaxisnucleus* (Bruguière, 1789) (Cerithioidea, Planaxidae) is a small, gregarious gastropod that lives in moderate to large population densities in intertidal, well-oxygenated habitats throughout the Caribbean. It occurs on hard substrates, from small pebbles and cobbles to large boulders or massive bedrock (Vermeij 1973; Bandel 1976; Houbrick 1987), from which it presumably grazes the biofilm. Individuals remain concealed from the sun under spray-moistened rocks, but emerge to feed, migrating with the tide (Bandel 1976). Rather little has been published on its biology, despite its ubiquity in the Caribbean. Similar to other planaxids, the species broods its young in a subhemocoelic brood pouch (Simone 2001; Strong et al. 2011), which are released from a pore on the side of the neck at the veliger stage (Thorson 1940; Bandel 1976; Houbrick 1987). Bandel (1976) observed freshly collected females to release veligers throughout the year. Troschel (1858) and Bandel (1984) provided descriptions of the radula of the species, and Houbrick (1987) and Simone (2001) described the radula and anatomy.

Following the serendipitous discovery that syntopic specimens from Guadeloupe clustered in two molecular groups, freshly collected material from Curaçao and Barbados confirmed the existence of two molecular clades within *Supplanaxisnucleus*. Museum material was then examined to evaluate the global distribution of the two clades.

In the present paper, we re-assess the taxonomy of *Supplanaxisnucleus*; we stabilize its nomenclature through the fixation of a neotype and review the nominal planaxid species currently treated as synonyms; and we attribute the second, molecularly distinct taxon to the little-known *S.nancyae* (Petuch, 2013). Finally, we remove *Planaxisnucleola* Mörch, 1876 from the synonymy of *S.nucleus*, and revalidate it as a species of *Hinea* Gray, 1847.

## Materials and methods

Specimens for molecular and morphological study were collected intertidally from three sites in the Lesser Antilles (Guadeloupe, Curaçao, Barbados); tissues were separated from the shells following flash-boiling or microwaving (Fukuda et al. 2008; Galindo et al. 2014) and preserved in 95% EtOH.

Radulae were tissue digested overnight in 100 µl of ATL lysis buffer (Qiagen, Inc.) containing ~ 50 µg of Proteinase-K, sonicated and rinsed in de-ionized water (Holznagel 1997). Cleaned radulae were mounted on aluminum stubs using carbon adhesive tabs, then coated with 25–30 nm gold/palladium (60/40) and photographed using an Apreo scanning electron microscope (FEI Company) at the National Museum of Natural History. Shells were photographed using a Canon EOS 50D camera with a Canon EF 100 mm f/2.8 macro lens and Canon MT-24EX macro twin light flash (Canon USA, Inc.).

Whole genomic DNA was extracted from a ~ 1 mm^3^ tissue clip of the foot using an Autogenprep965 (Autogen, Holliston, MA) automated phenol:chloroform extraction with a final elution volume of 50 µL. A 691 base pair (bp) fragment of cytochrome *c* oxidase subunit I (COI) was amplified using the jgLCOI primer (Geller et al. 2013) in combination with Cerithioid_COIR (Strong and Whelan 2019); a 509–511 bp fragment of 16S ribosomal DNA was amplified with the universal 16SAR/BR primers (Palumbi et al. 1991). PCR reactions were performed with 1 µL of undiluted DNA template in 20 µL reactions. Reaction volumes for COI consisted of 10 µL of Promega Go-Taq Hotstart Master Mix, 0.15 µM each primer, 0.25 µg/µL BSA, 1.25% DMSO and an amplification regime of an initial denaturation at 95 °C for 7 min, followed by 45 cycles of denaturation at 95 °C for 45 s, annealing at 42 °C for 45 s, extension at 72 °C for 1 min and a final extension at 72 °C for 3 min. Reaction volumes for 16S were 1x Biolase (Bioline, Taunton, MA) reaction buffer, 500 µM dNTPs, 3 mM MgCl_2_, 0.15 µM each primer, 0.25 µg/µL BSA, 1 unit Biolase DNA polymerase and an amplification regime of initial denaturation at 95 °C for 5 min, followed by 35 cycles of denaturation at 95 °C for 30 s, annealing at 48 °C for 30 s and extension at 72 °C for 45 s, followed by a final extension at 72 °C for 5 min. PCR products were purified using the Exo-SAP-IT protocol (GE healthcare, Piscataway, NJ). BigDye 3.1 (ABI, Foster City, CA) sequencing reactions and sequencing on an ABI 3730XL DNA analyzer capillary array were done following manufacturer’s instructions.

Genes were sequenced in both directions to ensure accuracy. Chromatograms were visually inspected and corrected as necessary in Geneious Prime 2019 (Biomatters). COI alignments were translated into amino acids to check for stop codons and frameshift mutations, then trimmed to 658 bp representing the standard invertebrate barcoding region (Folmer et al. 1994). 16S sequences were aligned with MUSCLE (Edgar 2004) using default parameters as implemented in Geneious Prime. The final aligned length for the 16S dataset was 512 bp. All newly generated sequences have been deposited in GenBank (Table 1).

**Table 1.** Voucher registration numbers and GenBank accession numbers for sequenced specimens of *Supplanaxisnucleus* and *S.nancyae*. Sequences for *Planaxissulcatus*, *S.planicostatus*, and *S.niger* were downloaded from GenBank (source Wiggering et al. 2020).

Species	Voucher registration	Locality	COI	16S
***Planaxissulcatus***	ZMB 117933-1	Egypt, Hurghada	MT593025	MT587883
***Supplanaxisplanicostatus***	ZMB 108261-h1	Paitilla, Bay of Panama	–	MT621366
***Supplanaxisniger***	ZMB 117939-1	Madagascar, Southeast Island Ste Marie	MT587879	MT593021
	ZMB 117946-1	Indonesia, Sumatra, Aceh, Ule-le	MT587878	MT593020
***Supplanaxisnucleus***	MNHN-IM-2009-26685	Guadeloupe, Plage de Malendure	MT921856	MT921888
	MNHN-IM-2009-26686		MT921857	MT921889
	MNHN-IM-2009-26687		MT921858	MT921890
	MNHN-IM-2009-26688		MT921859	MT921891
	MNHN-IM-2019-1715		MT921860	MT921892
	MNHN-IM-2019-1716		MT921861	MT921893
	MNHN-IM-2019-1717		MT921862	MT921894
	MNHN-IM-2019-1718		MT921863	MT921895
	USNM 1618953	Curaçao, S shore, beach in front of CARMABI research station	MT921864	MT921896
	USNM 1618954		MT921865	MT921897
	USNM 1618955		MT921866	MT921898
	USNM 1618956		MT921867	MT921899
	MNHN-IM-2000-35804 (neotype)	Barbados, Hastings Rocks	MT921868	MT921900
	MNHN-IM-2019-1728		MT921869	MT921901
***Supplanaxisnancyae***	MNHN-IM-2009-26684	Guadeloupe, Plage de Malendure	MT921870	MT921902
	MNHN-IM-2019-1703		MT921871	MT921903
	MNHN-IM-2019-1704		MT921872	MT921904
	MNHN-IM-2019-1705		MT921873	MT921905
	MNHN-IM-2019-1706		MT921874	MT921906
	USNM 1618949	Curaçao, S shore, beach in front of CARMABI research station	MT921875	MT921907
	USNM 1618950		MT921876	MT921908
	USNM 1618951		MT921877	MT921909
	USNM 1618952		MT921878	MT921910
	MNHN-IM-2019-1729	Barbados, Hastings Rocks	MT921879	MT921911
	MNHN-IM-2019-1707		MT921880	MT921912
	MNHN-IM-2019-1708		MT921881	MT921913
	MNHN-IM-2019-1709		MT921882	MT921914
	MNHN-IM-2019-1710		MT921883	MT921915
	MNHN-IM-2019-1711		MT921884	MT921916
	MNHN-IM-2019-1712		MT921885	MT921917
	MNHN-IM-2019-1713		MT921886	MT921918
	MNHN-IM-2019-1714		MT921887	MT921919

Sequences of additional planaxids [*Supplanaxisniger* (Quoy and Gaimard, 1833), *Planaxisplanicostatus* G.B. Sowerby I, 1825] were downloaded from GenBank, including *Planaxissulcatus* (Born, 1778), which was used to root the tree. Phylogenetic reconstruction was conducted using Maximum Likelihood (ML) in IQ-TREE ver. 1.6.12 (Nguyen et al. 2015) as implemented on the IQ-TREE web server (Trifinopoulos et al. 2016). The best-fit partitioning scheme and the most appropriate substitution model for each partition were estimated using ModelFinder (Kalyaanamoorthy et al. 2017) and partition models (Chernomor et al. 2016). Nodal support was estimated with 1,000 ultra-fast bootstrap replicates (Hoang et al. 2018).

### Repositories

**FMNH**Field Museum of Natural History;

**MNHN** Muséum national d’Histoire naturelle, Paris;

**NHMUK**The Natural History Museum, London;

**USNM**National Museum of Natural History, Smithsonian Institution (formerly U.S. National Museum), Washington DC;

**ZMB**Museum für Naturkunde, Berlin;

**ZMK** Statens Naturhistoriske Museum, Copenhagen.

## Results

**Figure 1.**
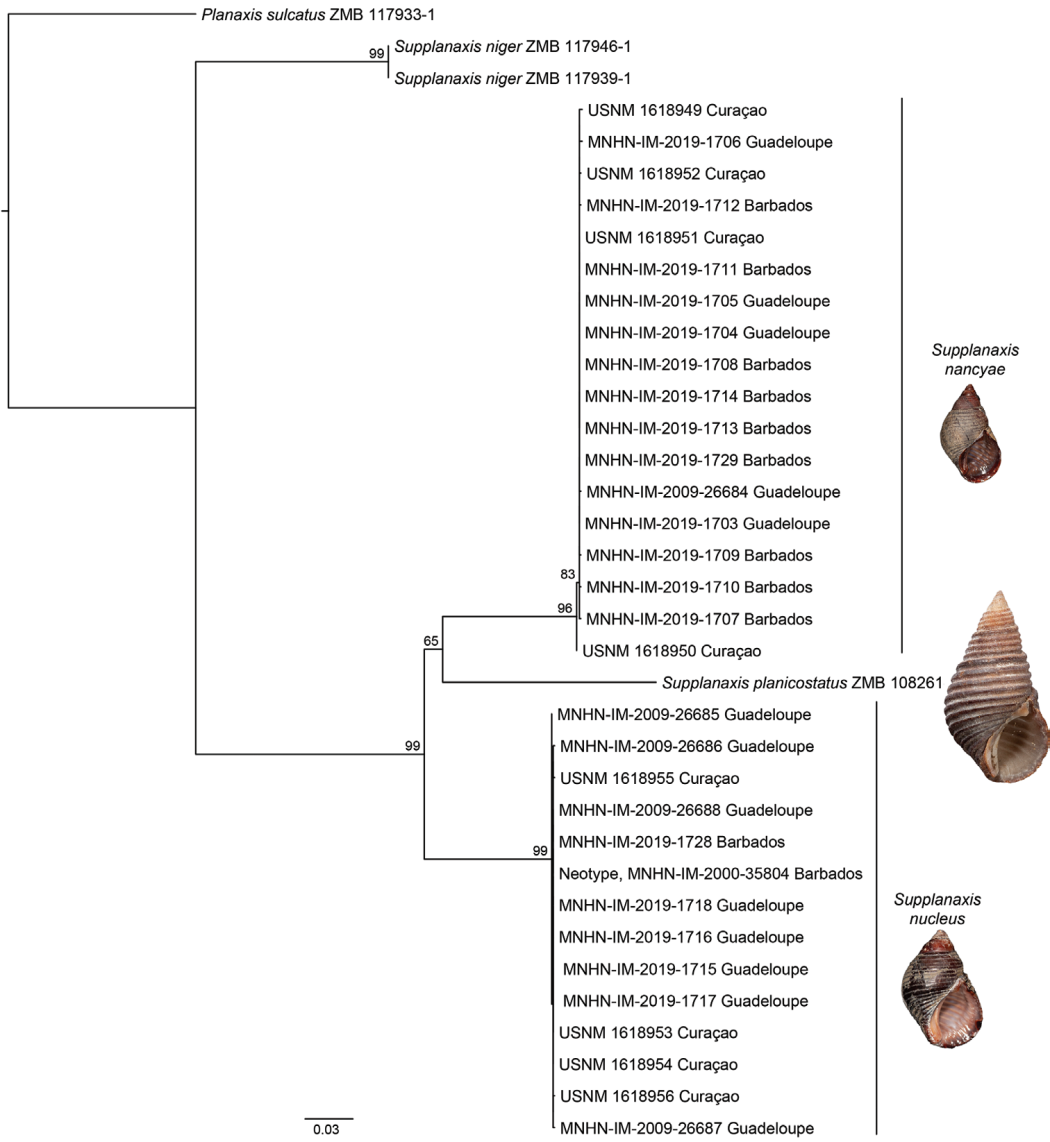
Phylogenetic tree produced via Maximum Likelihood using a concatenated alignment composed of partial mitochondrial COI and 16S sequences. ML bootstrap values greater than 50 are shown at the nodes. Figured specimens: *Supplanaxisnancyae*, MNHN-IM-2019-1729; *S.planicostatus*, syntype, NHMUK 1966623; *S.nucleus*, neotype, MNHN-IM-2000-35804.

Thirty-two individuals from three sites in Guadeloupe, Curaçao and Barbados were sequenced for portions of the COI and 16S mitochondrial genes (Table 1). The concatenated dataset was 1170 bp in length. The best-fit partitioning scheme used distinct models for each locus, with the best-fit model being MGK+F3X4 and HKY+F+G4 for COI and 16S, respectively. The ML tree constructed from the concatenated dataset (Fig. 1) resolved two strongly supported clades among what has been traditionally recognized as a single species, *Supplanaxisnucleus*. Individuals collected in syntopy from all three sites occurred in both clades, which differed by 11.6–12.2% uncorrected pairwise sequence divergence in COI, with no geographic structuring among sites. Examination of the shells and radula revealed diagnostic morphological features consistent with the recognition of two species.

### Systematics

**Class GASTROPODA Cuvier, 1795**

**Family PLANAXIDAE Gray, 1850**

#### Genus Supplanaxis

Taxon classificationAnimaliaCerithioideaPlanaxidae

Thiele, 1929

B106512D-CE00-5040-9FC1-76B14E36C5A4

Planaxis (Supplanaxis) Thiele, 1929. Type species: Buccinumnucleus Bruguière, 1789, by monotypy.
Planaxis (Proplanaxis) Thiele, 1929. Type species: Planaxisplanicostatus G.B. Sowerby I, 1825, by monotypy (syn. nov.).

##### Remarks.

Thiele (1929: 203) established *Supplanaxis* as a subgenus of *Planaxis*, for planaxids with rather small, dark colored, largely smooth shells, with spiral grooves mostly on the base of the last whorl and under the suture; and with a radula characterized by a broad rachidian with two to four denticles on either side, the long lateral extensions of the lateral teeth, and the outer marginal with a very broad, finely toothed cutting edge. Based on the morphological differences in shell, radula, soft anatomy, and embryonic development, Houbrick (1987) elevated *Supplanaxis* to full genus.

Thiele (1929: 203) also established *Proplanaxis* as a new subgenus of *Planaxis*, with *Planaxisplanicostatus* G.B. Sowerby I, 1825, as type species by monotypy. Houbrick (1987: 4) treated *Proplanaxis* as a synonym of *Planaxis*, but our molecular tree shows *P.planicostatus* to be nested within the *Supplanaxis* clade (see below), and not within *Planaxis* s.s., a taxonomic position in agreement with Laidre and Vermeij (2012), who had already used the combination *Supplanaxisplanicostatus*. The names *Supplanaxis* and *Proplanaxis* having been published simultaneously, we act as First Revisers and, under Art. 24.2 of the ICZN Code, give precedence to the name *Supplanaxis* over *Proplanaxis*.

#### Supplanaxis nucleus

Taxon classificationAnimaliaCerithioideaPlanaxidae

(Bruguière, 1789)

44815B04-DCCE-5AB2-99E8-3088FEEA22B9

**Figure 2.**
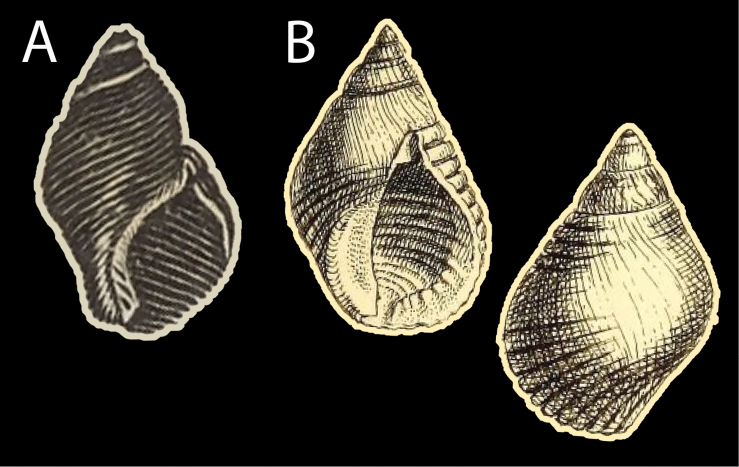
Original figures **A***Buccinumnucleus* Bruguière, 1789 (Lister 1770: pl. 976, fig. 32) **B***Planaxissemisulcatus* G.B. Sowerby I, 1823 ([pl. 73], fig. 3).

**Figure 3.**
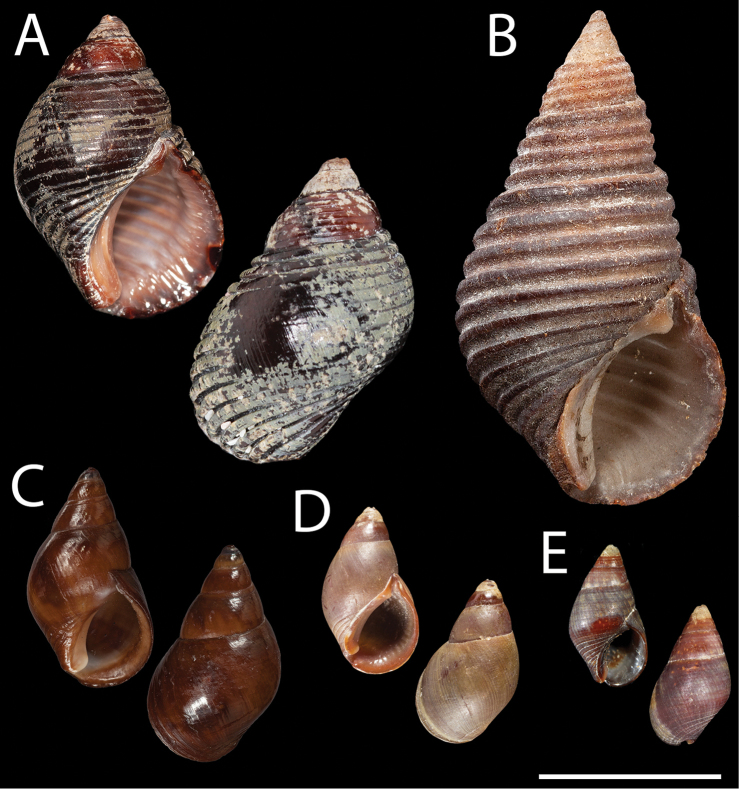
Type specimens of *Supplanaxisnucleus* and relevant planaxids. **A***Buccinumnucleus* Bruguière, 1789. Neotype, MNHN-IM-2000-35804 **B***Planaxisplanicostatus* G.B. Sowerby I, 1825. Syntype NHMUK 1966623 (© The Trustees of the Natural History Museum, London, http://creativecommons.org/licenses/by/4.0/; https://data.nhm.ac.uk/object/4767541c-a5d1-41db-aa0c-0c03724e970a) **C**Planaxis (Supplanaxis) nancyae Petuch, 2013. Holotype, FMNH 328402 (© Field Museum of Natural History–CC BY-NC; https://collections-zoology.fieldmuseum.org/catalogue/2877223) **D***Planaxisniger* Quoy and Gaimard, 1833. Syntype, one of 22 specimens, MNHN-IM-2000-27769 **E***Planaxisnucleola* Mörch, 1876. Probable holotype, ZMK 152749. Scale bar: 1 cm.

**Figure 4.**
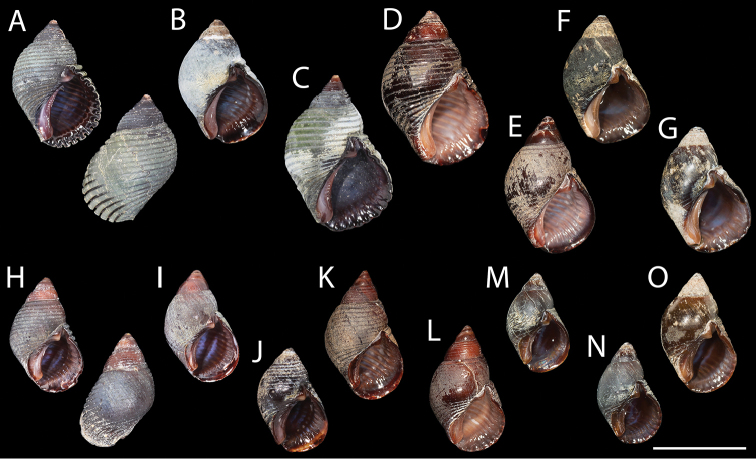
Shell morphology of *Supplanaxis* from the Caribbean. Sequenced vouchers **A–G***S.nucleus***H–O***S.nancyae***A–C** Guadeloupe, Plage de Malendure **A**MNHN-IM-2019-1716 **B**MNHN-IM-2009-26686 **C**MNHN-IM-2009-26687 **D–E** Barbados, Hastings Rocks. **D** Neotype, MNHN-IM-2000-35804 **E**MNHN-IM-2019-1728 **F–G** Curaçao, S shore, beach in front of CARMABI research station **F**USNM 1618956 **G**USNM 1618953 **H–J** Guadeloupe, Plage de Malendure **H**MNHN-IM-2019-1703 **I**MNHN-IM-2019-1704 **J** IM MNHN-2009-26684 **K–L** Barbados, Hastings Rocks **K**MNHN-IM-2019-1729 **L**MNHN-IM-2019-1711 **M–O** Curaçao, S shore, beach in front of CARMABI research station **M**USNM 1618949 **N**USNM 1618951 **O**USNM 1618952. Scale bar: 1 cm.

**Figure 5.**
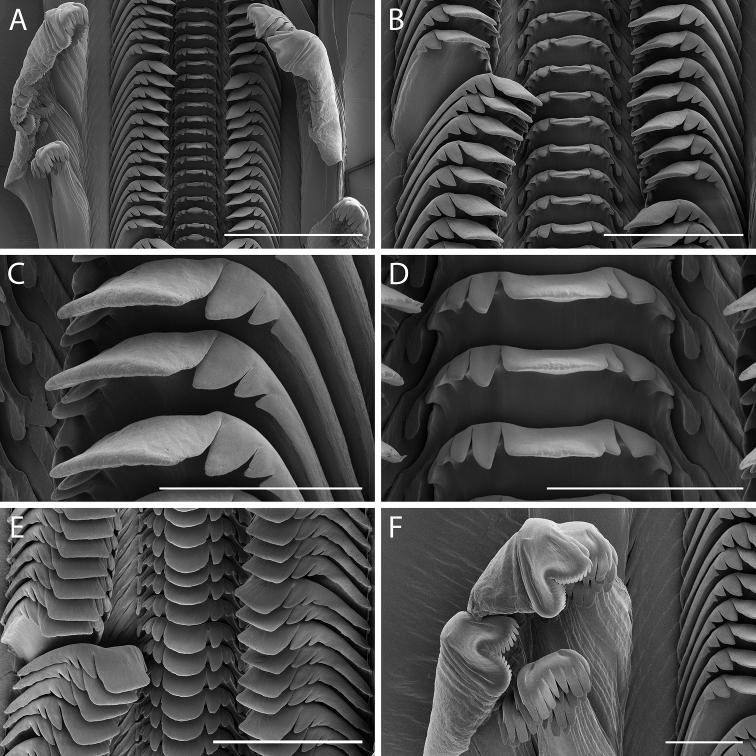
Radular morphology of *Supplanaxisnucleus* (MNHN-IM-2019-1716) **A** Radular ribbon **B** Rachidian and lateral teeth **C** Detail of lateral teeth **D** Detail of rachidian **E** Rachidian and lateral teeth viewed at a ~ 45° angle from above, showing detail of cutting edge. **F** Internal and external lateral teeth. Scale bars: 200 µm (**A**), 100 µm (**B, E**), 50 µm (**C, D, F**).

Figs 2 , 3A , 4A–G , 5 , 7A–L at right

Buccinum nucleus Bruguière, 1789: 211–212
Planaxis semisulcatus G.B. Sowerby I, 1823: [pl. 73] fig. 3

##### Neotype.

BARBADOS • 16.9 mm in height; Hastings Rocks; 13°04'25"N, 59°35'39"W, Dec. 2018; P Bouchet leg.; MNHN-IM-2000-35804, here designated; GenBank: MT921868, MT921900 (Figs 3A, 4D, 7L at right).

Bruguière (1789: 254–255) based his description of *Buccinumnucleus* on three sources: (i) an illustration of a shell from Barbados published by Lister (1770: pl. 976 fig. 32) (Fig. 2A); (ii) specimens collected by himself at Foule-Pointe in Madagascar; (iii) shells used in a decorated garment brought back from New Zealand by Cook.

(i) The shell from Barbados was accompanied by a non-binominal legend *Buccinum B.r.paruum nigrum, ex toto laeve* and the locality Barb., which was rendered by Bruguière as *Buccinum brevirostrum parvum nigrum extoto laeve Barbadense*.

Some of the Lister collection was acquired by Sloane, and the Sloane collection was one of the founding collections of the British Museum (Natural History) [now The Natural History Museum], in London. However, there is no material in NHMUK corresponding to Lister’s illustration (Wilkins 1953; and A. Salvador, pers. comm.), and this specimen is to be considered lost.

(ii) Bruguière was a member of the second expedition (1773–1774) led by Kerguelen to the Subantarctic islands that were later to be called Kerguelen Is. On the return journey, his ship called in February-March 1774 at Foulpointe (or “Foule-Pointe” as spelled by Bruguière), now Mahavalona [district of Toamasina (Tamatave)] on the east coast of Madagascar. It is not known which, if any, natural history collections Bruguière brought back to France. He (Bruguière 1789: 255) used the past tense to refer to the specimens collected at Foule-Pointe “he had owned previously”. Regardless, there are no traces of this material in MNHN.

(iii) Bruguière additionally recorded *Buccinumnucleus* based on specimens used for decoration on garments brought back by Cook from New Zealand, that Bruguière had seen in the cabinet of Mr Broussonet, then Secretary of the (French) Royal Society of Agriculture. Pierre Marie Auguste Broussonet (1761–1807) was a French scientist who was based in London in 1780, where he met such scientists as Banks, Forster and Solander. It is therefore possible that Broussonet could have acquired Cook artifacts from this circle and brought them back with him to Paris. He took part in the French Revolution but, as a member of the “Girondins”, had to leave Paris in 1793 and his belongings were seized. However, Broussonet’s artifacts are not nowadays traceable in any French museum where they would have been deposited by the Revolutionary powers. Dr Adrienne Kaeppler, curator for the Pacific Islands in the Department of Anthropology at the Smithsonian’s National Museum of Natural History, has advised us that Maori cloaks from Cook’s voyage to New Zealand are not known to be decorated with shells, and a Tonga or Hawaii provenance for Broussonet’s artifacts would have been more likely.

There are thus no specimens left that could be considered a syntype of *Buccinumnucleus*. The specimens from Madagascar or the Pacific would not have been conspecific with the shell from the Caribbean illustrated by Lister; they might have been *Supplanaxisniger* (Quoy and Gaimard, 1833) (Fig. 3D), which bears a strong resemblance to *S.nucleus*–especially to the eye of an 18^th^ century conchologist.

Because the description of *Buccinumnucleus* referred to more than one species, and because the Caribbean species designated under that name is a complex of two cryptic species, it is desirable to stabilize the nomenclature by the fixation of a neotype, which we designate herein.

*Planaxissemisulcatus* G.B. Sowerby I, 1823 (1823: [pl. 73], fig. 3) (Fig. 2B) was described from an unknown locality. It has been treated as a synonym of *Supplanaxisnucleus* since at least Smith (1872: 40), an opinion accepted by Sowerby II (1877: pl. 1, species no. 7; 1884: 177). There is no known type material in NHMUK (A. Salvador, pers. comm.). To stabilize the nomenclature, we are here designating the neotype of *Buccinumnucleus* also as the neotype of *Planaxissemisulcatus*.

##### Other material.

ANTIGUA AND BARBUDA • 1 spm; Antigua, Green Island; [17°04'12"N, 61°40'08"W]; 24 Apr. 1958; Smithsonian - Bredin Caribbean Expedition; R/V Freelance; J Clarke leg.; USNM 738725.

BAHAMAS • 1 spm; Rawson leg.; USNM 54705; • 1 spm; New Providence, The Caves; [25°04'10"N, 77°27'02"W]; H Dodge leg.; USNM 603895.

BARBADOS • 1 spm; Hastings Rocks; 13°04'25"N, 59°35'39"W; Nov. 2018; P Bouchet leg.; GenBank: MT921869, MT921901; MNHN-IM-2019-1728.

BELIZE • 2 spms; Near Carrie Bow Cay; 27 Apr. 72; CCRE - Caribbean Coral Reef Ecosystems, Belize; RS Houbrick leg.; reef flat on algal covered rocks along shore; USNM 770861; • 18 spms; Carrie Bow Cay, NE Cay, Glover’s Reef; 15 Aug. 1973; CCRE - Caribbean Coral Reef Ecosystems, Belize; RS Houbrick leg.; intertidal on rocks; USNM 771083; • 5 spms; Carrie Bow Cay; depth 0.5 m; 12 Jan. 1976; CCRE - Caribbean Coral Reef Ecosystems, Belize; windward, reef crest, reef flat; USNM 828774.

BRITISH VIRGIN ISLANDS • 1 spm; Tortola, Newman Island, Treasure Point; 6 Apr. 1958; Smithsonian - Bredin Caribbean Expedition; R/V Freelance; shore coll.; USNM 683319; • 1 spm; Guana Island, White Bay; Smithsonian - Bredin Caribbean Expedition; R/V Freelance; W Schmitt leg.; from reefs in cove; USNM 735906.

COLOMBIA • 3 spms; Carthagena; Chamberlain leg.; USNM 131448; • 1 spm; Vicinity of Carthagena; JA Link leg.; USNM 364399; • 1 spm; NE of Santa Marta; 12 Oct. 1977; M Jones leg.; assoc. with flat intertidal and barely subtidal rocks; USNM 770120.

COSTA RICA • 22 spms; Limón Province, Portete; 11 Jul. 1966; RS Houbrick leg.; common on rocks; USNM 702828; • 14 spms; Limón Province, Playa Bonita; 10 Mar. 1965; RS Houbrick leg.; on rocks, low tide; USNM 702839; • 1 spm; Limón Province, Cahuita, Portete; 13 Jul. 1964; D Huckebey leg.; USNM 702859; • 19 spms; Limón Province, Portete; 11 Jul. 1966; RS Houbrick leg.; USNM 706451.

CUBA • 1 spm; Guantanamo, Cable House; Tomas Barrera Expedition; Schooner Tomas Barrera; J Henderson and P Bartsch leg.; shore; USNM 450605; • 3 spms; Cardenas, Peninsula de Hicacos; V Conde leg.; USNM 599927; • 2 spms; Punta de Maya, in front of lighthouse; 13 Nov. 80; Cuba Expedition 1980; J Rosewater leg.; dogtooth coral; USNM 803412; • 1 spm; Veradero, La Conchito; 14 Nov. 80; Cuba Expedition 1980; J Rosewater leg.; USNM 803413; • 1 spm; Cojimar; 15 Nov. 80; J Rosewater leg.; USNM 807614.

CURAÇAO • 1 spm; S shore, beach in front of CARMABI research station; 12°07'20"N, 68°58'08"W; 18 May 2015; E Strong leg.; among cobbles; GenBank: MT921864, MT921896; USNM 1618953; • 1 spm; ibid; GenBank: MT921865, MT921897; USNM 1618954; • 1 spm; ibid; GenBank: MT921866, MT921898; USNM 1618955; • 1 spm; ibid; GenBank: MT921867, MT921899; USNM 1618956.

DOMINICAN REPUBLIC • 2 spms; Oro Oro Beach [sic, possibly Playa Dorada], S. shore; [19°46'27"N, 70°38'30"W]; H Dodge leg.; USNM 603900.

GUADELOUPE • 1 spm; Aug. 1946; on rocks; USNM 487939; • 1 spm; Plage de Malendure; 16°10'28"N, 61°46'47"W; 7 May 2012; KARUBENTHOS Expedition stn. GM7; GenBank: MT921856, MT921888; MNHN-IM-2009-26685; • 1 spm; ibid; GenBank: MT921857, MT921889; MNHN-IM-2009-26686; • 1 spm; ibid; GenBank: MT921858, MT921890; MNHN-IM-2009-26687; • 1 spm; ibid; GenBank: MT921859, MT921891; MNHN-IM-2009-26688; • 1 spm; Plage de Malendure; 16°10'28"N, 61°46'47"W; 1 Apr. 2017; D Lamy leg.; GenBank: MT921860, MT921892; MNHN-IM-2019-1715; • 1 spm; ibid; GenBank: MT921861, MT921893; MNHN-IM-2019-1716; • 1 spm; ibid; GenBank: MT921862, MT921894; MNHN-IM-2019-1717; • 1 spm; ibid; GenBank: MT921863, MT921895; MNHN-IM-2019-1718.

HAITI • 130 spms; Orcutt leg.; USNM 383065; • 6 spms; Anse à Cochons; R/V Eolis; J Henderson leg.; shore; USNM 434880; • 150 spms; Département de l’Ouest, Saltron; Orcutt leg.; USNM 439970; • 11 spms; Département du Sud, Port-Salut; Orcutt leg.; USNM 440004; • 2 spms; Forban; A Curtiss leg.; USNM 518264.

HONDURAS • 26 spms; Swan Island; Townsend leg.; USNM 83649; • 41 spms; Utilla Island; [16°05'47"N, 86°55'44"W]; Simpson leg.; USNM 434879; • 2 spms; Webb leg.; USNM 434885.

JAMAICA • 28 spms; St. Thomas, Morant Bay; Orcutt leg.; USNM 401450; • 4 spms; Pt. Antonio; CWJ leg.; USNM 434882; • 24 spms; Annotta Bay; Orcutt leg.; USNM 440695; • 17 spms; Portland, Port Antonio; Orcutt leg.; USNM 440864; • 15 spms; Portland, near Buff Bay; Orcutt leg.; USNM 441213; • 2 spms; Montego Bay; Mrs LS McLean leg.; USNM 464162; • 8 spms; Portland, Port Antonio; Vendeyes, Orcutt leg.; USNM 518050; • 1 spm; Montego Bay Area, Doctor’s Cave Pier; 26 Nov. 1976; Paul and Sharon Greenhall leg.; USNM 767665; • 6 spms; St. Mary, 2 mi. N Port Maria; 18°24'19"N, 76°53'18"W; JI McCurkin leg.; 1 ft above splash line under cobbles; USNM 770590.

MEXICO • 79 spms; Quintana Roo, Mujeres Island, 0.5 mi. S of village; 29 Mar. 1960; Smithsonian - Bredin Caribbean Expedition; R/V Blue Goose; E Bousfield, H Rehder and W Schmitt leg.; rocky open ocean shore; USNM 662233; • 27 spms; Cozumel, 1 mi. N of San Miguel, in front of Cabanas del Caribe property; [20°31'59"N, 86°56'21"W]; 3 Apr. 1960; Smithsonian - Bredin Caribbean Expedition; R/V Blue Goose; H Rehder, W Schmitt and E Bousfield leg.; on rocks from above high tide to below water line; USNM 662773; • 3 spms; 100 km. SSE Tampico, Lobos Reef; depth 0-6 ft; 5 Jun. 1973; JW Tunnell leg.; coral spoil, along ship channel; USNM 710350; • 4 spms; Quintana Roo, Cozumel Island, San Miguel, along shore 0.25 NE of pier; 29 Apr. 1960; Smithsonian - Bredin Caribbean Expedition; R/V Blue Goose; W Schmitt leg.; on rocks; USNM 736539; • 1 spm; Quintana Roo Ids., Cozumel, N Pt., nr. Lighthouse; depth 6 ft; 9 Apr. 1960; Smithsonian - Bredin Caribbean Expedition; R/V Blue Goose; H Rehder and E Bousfield leg.; coral rocks, splash pools; USNM 736750.

MONTSERRAT • 3 spms; Fox Bay, just N of Plymouth; 20 Apr. 1959; Smithsonian - Bredin Caribbean Expedition; R/V Caribee; black sand beach; USNM 682493.

NETHERLANDS ANTILLES • 6 spms; St. Martin; Ford leg.; USNM 434878.

PANAMA • 4 spms; Colón; Stearns leg.; USNM 54700; • 10 spms; San Blas; TG Thompson leg.; USNM 597416; • 33 spms; Puerto Perme, NW of Cape Tiburon; [8°44'13"N, 77°32'41"W]; 14 Mar. 1953; RH Stewart leg.; USNM 664216; • 11 spms; 3rd cove SW of Buenaventura; 19 Apr. 1971; Smithsonian STRI Panama Survey; on cobbles on cobble beach with coral heads and tide pools; USNM 732810; • 30 spms; 1.7 km WSW of Portobelo, on bay side of Coral Pt.; 11 Nov. 1971; Smithsonian STRI Panama Survey; large cobbles, boulders; USNM 734007; • 1 spm; 1.7 km WSW of Portobelo, on bay side of Coral Pt.; 11 Nov. 1971; Smithsonian STRI Panama Survey; fauna assoc. with rocks; USNM 734020; • 1 spm; Canal Zone, Ft. Sherman, Toro Pt., outside base of jetty; 13 Nov. 1971; Smithsonian STRI Panama Survey; under cobbles; USNM 734070; • 5 spms; Canal Zone, Galeta Island; 23 Apr. 1972; Smithsonian STRI Panama Survey; varied habitat; USNM 734717; • 1 spm; Canal Zone, Ft. Sherman, Punta De Toro; 25 Apr. 1972; Smithsonian STRI Panama Survey; Thalassia and hard limestone substrate with potholes; USNM 734763; • 1 spm; Canal Zone, Gatun Locks, lower W chamber; Intertidal; 20 Mar. 1972; Smithsonian STRI Panama Survey; at water edge outside of casson; USNM 742409; • 5 spms; San Blas, Pico Feo; 8 Nov. 1972; Smithsonian STRI Panama Survey; USNM 743277.

PUERTO RICO • 11 spms; Fajardo; 10 Dec. 1951; J Weber leg.; USNM 663091; • 3 spms; USNM 1291138.

ST. VINCENT AND THE GRENADINES • 6 spms; St. Vincent; Chamberlain leg.; USNM 131804.

TRINIDAD AND TOBAGO • 1 spm; Trinidad, Chaguaramas Bay; SP Archino leg.; USNM 518500; • 5 spms; Trinidad, N.A.S., Macqueripe Beach; CI Aslaksan leg.; USNM 608788; • 1 spm; Tobago; 5 Nov. 1953; J Weber leg.; USNM 663352; • 2 spms; Tobago, Buccoo Reef; 5 Apr. 1959; Smithsonian - Bredin Caribbean Expedition; R/V Caribee; USNM 682203.

U.S. VIRGIN ISLANDS • 3 spms; St. Thomas; CB Adams leg.; USNM 6419; • 213 spms; St. Thomas, edge Magens Bay; Shoemaker leg.; rocks; USNM 214816; • 3 spms; Water Island, Drift Bay; Shoemaker leg.; USNM 214882; • 5 spms; St. Thomas; M Petit leg.; USNM 250143; • 143 spms; St. Thomas; Petit leg.; USNM 530351; • 2 spms; St. Croix; 10 Sep. 1956; J Weber leg.; USNM 663480; • 5 spms; St. Thomas, S side Careen Hill; M Jones leg.; rocky intertidal; USNM 666124; • 1 spm; St. Thomas, S end Magens Bay; M Jones leg.; rocks adjacent to beach; USNM 666146; • 58 spms; St. Thomas, S end Magens Bay; M Jones leg.; rocks adjacent to beach; USNM 666148; • 2 spms; St. John, Francis Bay, McCauley beach; 28 Mar. 1958; Smithsonian - Bredin Caribbean Expedition; R/V Freelance; from rocks; USNM 683466; • 2 spms; St. Croix; W Old leg.; USNM 714103; • 11 spms; St. John, Caneel Bay; [18°20'34"N, 64°47'13"W]; 25 Mar. 1958; Smithsonian - Bredin Caribbean Expedition; R/V Freelance; C Shuster and J Finlay leg.; USNM 738775.

UNITED STATES • 6 spms; Miami; Offer leg.; USNM 159423; • 2 spms; Palm Beach Inlet; Ted Bayer leg.; under jetty rocks; USNM 513679; • 1 spm; Tortugas, S of Long Key; USNM 780137; • 27 spms; Miami Beach, South Beach, jetty at inlet; 21 Apr. 82; RS Houbrick leg.; in rubble at intertidal zone; USNM 809779; • 30 spms; Miami Beach, S Miami Beach, jetties; 16 Mar. 83; RS Houbrick leg.; intertidal, on and under rocks.; USNM 809780; • 4 spms; Miami Beach, South Beach, at jetty; [25°45'48"N, 80°07'45"W]; 12 Aug. 84; RS Houbrick leg.; intertidal rock and boulders; USNM 842270; • 18 spms; Miami Beach, South Beach, at jetty; [25°45'48"N, 80°07'45"W]; 5 Sep. 83; RS Houbrick leg.; among intertidal boulders, high tide mark; USNM 842271; • 12 spms; Miami Beach, South Beach, at jetty; [25°45'48"N, 80°07'45"W]; 20 Nov. 83; RS Houbrick leg.; intertidal, on rocks and boulders; USNM 842272; • 20 spms; South Miami Beach; 16 Jan. 1984; RS Houbrick leg.; USNM 858456; • 17 spms; Palm Beach Inlet, South jetty; 14 Jan. 39; F Bayer leg.; under stones; USNM 889605; • 14 spms; offshore rocks off south central edge of Boca Chica Key; 26 Feb. 1975; P Poland leg.; under intertidal rocks near low water mark; USNM 1446615.

VENEZUELA • 31 spms; Macuto; Lyon and Robinson leg.; rocks along shore; USNM 170221; • 6 spms; Sucre, Pena, Manacuare; Arnold leg.; USNM 252635.

##### Description.

*Shell.* Shell (Figs 3A, 4A–G, 7A–L at right) large for the genus, solid, littoriniform, medium high-spired, consisting of 5+ (apex generally broken or corroded in adults) moderately convex whorls separated by impressed suture, last whorl occupying ca 75–82% of total shell height. Shell surface usually covered with 15–17 even, deeply incised spiral grooves extending over all shell height, especially well marked adapically and on base behind outer lip. Aperture ovoid, expanding abaxially at mid-height, occupying ca 50% of total shell height, with 7–10 sharp internal lirae, columellar pillar extending almost to the abapical point of the aperture, delimiting a narrow siphonal notch, callus adpressed to parietal and columellar areas, parietal tooth strong, blunt. *Color* uniformly reddish brown to dark violet, parietal tooth and columellar callus orange violet. *Height* 11–18 mm.

*Neotype* (Figs 3A, 4D, 7L at right) reddish brown, with weak parietal tooth, height 16.9 mm.

*Radula*. Radula taenioglossate (Fig. 5A). Rachidian pentagonal, with broad basal plate, long basolateral extensions, and short, rounded, median basal projection (Fig. 5B, D). Upper lateral part of basal plate with narrowly rectangular basal denticle. Rachidian cutting edge broad, comprising two-thirds width of tooth. Cutting edge shallowly and smoothly concave with large, squarish, spatulate median cusp, flanked by three to four smaller pointed denticles on each side. Lateral tooth with broad, high basal plate with central supporting ridge, rounded margins, and long lateral basal extension (Fig. 5C). Cutting edge broad with large, squarish, central cusp flanked by three to four inner and two to three sharp outer denticles. Marginal teeth elongate with curved paw-like tips (Fig. 5A, F). Inner marginal tooth with narrow flange along length of shaft outer edge, and with concave, rake-like tip with nine to ten rounded denticles. Outer marginal tooth with broad, membranous flange along distal outer edge, and with broad, bilobed tip bearing approximately 28 to 30 small, rounded denticles.

**Figure 6.**
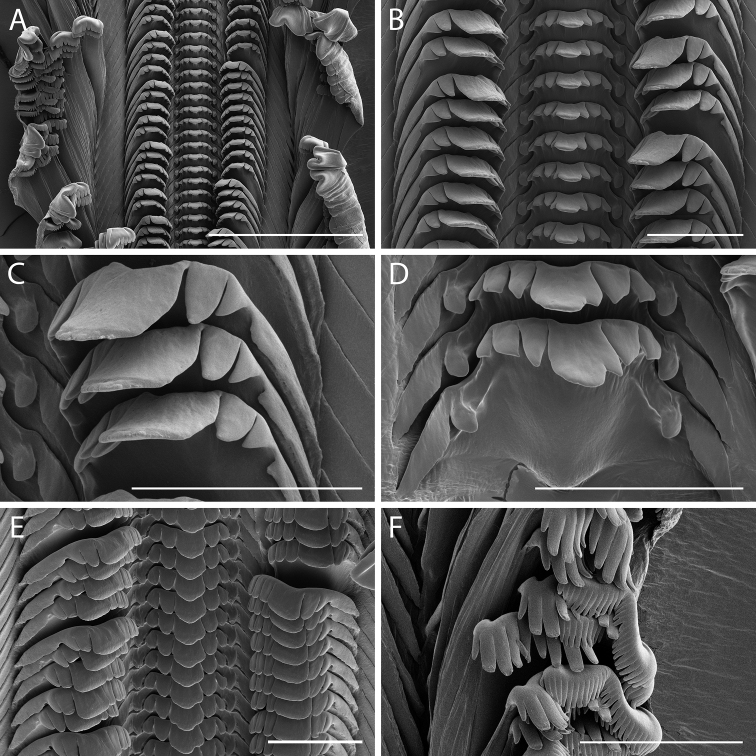
Radular morphology of *Supplanaxisnancyae* (MNHN-IM-2019-1703, except as noted) **A** Radular ribbon **B** Rachidian and lateral teeth **C** Detail of lateral teeth **D** Detail of rachidian. Note fusion of innermost denticle with central cusp **E** Rachidian and lateral teeth viewed at a ~ 45° angle from above, showing detail of cutting edge **F** Internal and external lateral teeth (MNHN-IM-2019-1704). Scale bars: 200 µm (**A**), 50 µm (**B, C, D, E, F**).

##### Distribution and ecology.

**Figure 7.**
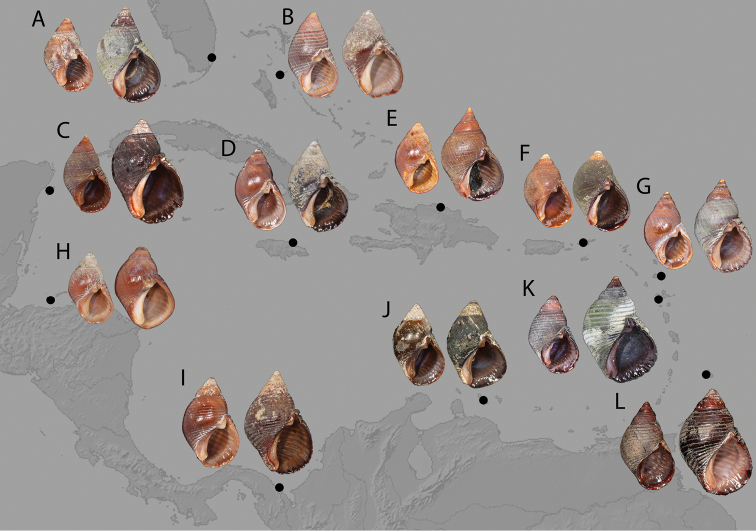
Comparative shell morphology of *Supplanaxis* around the Caribbean. *Supplanaxisnancyae* is shown at left, and *S.nucleus* is at right, for each pair. The individuals from each pair were sampled from the same site and were originally part of the same lot **A** Florida, Miami Beach, South Beach, at jetty (at left, USNM 1620287; at right, USNM 842270) **B** Bahamas, New Providence, The Caves (at left, USNM 1620274; at right, USNM 603895) **C** Mexico, Cozumel, 1 mi. N of San Miguel (at left, USNM 1620276; at right, USNM 662773) **D** Jamaica, St. Mary, 2 mi. N Port Maria (at left, USNM 1620285; at right, USNM 770590) **E** Dominican Republic, Oro Oro Beach [sic, possibly Playa Dorada], S. shore (at left, USNM 1620275; at right, USNM 603900) **F** Virgin Islands, St. John, Caneel Bay (at left, USNM 1620284; at right, USNM 738775) **G** Antigua and Barbuda, Antigua, Green Id. (at left, USNM 1620283; at right, USNM 738725) **H** Honduras, Utilla Id. (at left, USNM 1620264; at right, USNM 434879) **I** Panama, Puerto Perme, NW of Cape Tiburon (at left, USNM 1620279; at right, USNM 664216) **J** Curaçao, S shore, beach in front of CARMABI research station (at left, USNM 1618952; at right USNM 1618956; sequenced vouchers) **K** Guadeloupe, Plage de Malendure (at left, MNHN-IM-2019-1703; at right MNHN-IM-2009-26687; sequenced vouchers) **L** Barbados, Hastings Rocks (at left, MNHN-IM-2019-1729; at right, neotype, MNHN-IM-2000-35804; sequenced vouchers). Base map: Wikimedia Commons contributors (2017).

Throughout the Caribbean in high energy, intertidal environments, on hard substrates, from large boulders to small cobbles and pebbles, in populations of moderate to large size (Vermeij 1973; Bandel 1976; Houbrick 1987). Its range extends from Palm Beach Inlet, Florida, in the north, to the northern coast of South America, from Veracruz, Mexico in the west (Tunnell 1974) and as far east as Trinidad and Tobago off Venezuela, including the Gulf of Mexico, Caribbean Sea and the Antillean Arc (Fig. 7). It is “rare” in Bermuda (Sterrer and Schoepfer-Sterrer 1986: 413), which may indicate that it only forms pseudopopulations there, and is absent from the Guyanas and Brazil.

##### Remarks.

It is evident, from the morphology of the rachidian, that the radular descriptions of Troschel (1858: pl. 12, fig. 9A–D), Houbrick (1987: fig. 18A–E) and Simone (2001: fig. 88) were based on *S.nucleus*. Houbrick (1987: figs 19A, B, 20A–I, 21A–G, 22A–F) described the external anatomy and gross morphology of the mantle cavity, alimentary tract, and excretory, reproductive and nervous systems based on specimens from the same lot that yielded the radula (USNM 809780). Examination of the shells from this lot confirmed them all to be *S.nucleus*, although other lots collected from the same site at subsequent occasions comprised mixtures of the two species. Simone (2001: figs 56, 74, 88, 189–206) also provided a detailed description of the anatomy based on material obtained from several sites in Venezuela. The figured shells (figs 17–19) are *S.nucleus*, but both species occur in Venezuela and it is possible the anatomical descriptions are composite.

Despite its abundance in modern-day Caribbean faunas, *Supplanaxisnucleus* is surprisingly recorded as a fossil only in the Upper Pleistocene of Venezuela (Weisbord 1962: 168, pl. 14, figs 17, 18; and B. Landau, pers. comm.). It is not known from the well-preserved horizons of Florida.

#### Supplanaxis nancyae

Taxon classificationAnimaliaCerithioideaPlanaxidae

(Petuch, 2013) comb. nov.

FB5B7C64-64E4-5B62-81AD-61ACEBC88F2C

Figs 3C , 4H–O , 6 , 7A–L at left

Planaxis (Supplanaxis) nancyae Petuch, 2013: 193, fig. 6.13A, B

##### Holotype.

HAITI • 11 mm in height; off southern Gonave Island; depth 2 m; under rocks; FMNH 328402 (Fig. 3C)

##### Other material.

ANGUILLA • 6 spms; 29 Apr. 1958; Smithsonian - Bredin Caribbean Expedition; USNM 738885?

ANTIGUA AND BARBUDA • 9 spms; Antigua, Shell Beach; CI Aslaksan leg.; USNM 608782; • 2 spms; Antigua, Nonsuch Bay, N side of Bird Island; depth 4 ft; 24 Apr. 1958; Smithsonian - Bredin Caribbean Expedition; R/V Freelance; shore coll.; USNM 683510; • 21 spms; Redonda Island, lee side; 10 Apr. 1956; Smithsonian - Bredin Caribbean Expedition; R/V Freelance; W Schmitt leg.; wave washed rocks; USNM 714035; • 67 spms; Antigua, Green Island; [17°04'12"N, 61°40'08"W]; 24 Apr. 1958; Smithsonian - Bredin Caribbean Expedition; R/V Freelance; J Clarke leg.; USNM 1620283 (ex USNM 738725).

BAHAMAS • 5 spms; Rawson leg.; USNM 1620260 (ex USNM 54705); • 2 spms; New Providence, The Caves; [25°04'10"N, 77°27'02"W]; H Dodge leg.; USNM 1620274 (ex USNM 603895).

BARBADOS • 1 spm; USNM 19768; • 1 spm; EW Williams, CB Lungren leg.; USNM 341803; • 4 spms; beach; USNM 459982; • 32 spms; off Needham Pt.; low tide; USNM 459983; • 1 spm; Hastings Rocks; 13°04'25"N, 59°35'39"W; Nov. 2018; P Bouchet leg.; GenBank: MT921879, MT921911; MNHN-IM-2019-1729; • 1 spm; ibid; GenBank: MT921880, MT921912; MNHN-IM-2019-1707; • 1 spm; ibid; GenBank: MT921881, MT921913; MNHN-IM-2019-1708; • 1 spm; ibid; GenBank: MT921882, MT921914; MNHN-IM-2019-1709; • 1 spm; ibid; GenBank: MT921883, MT921915; MNHN-IM-2019-1710; • 1 spm; ibid; GenBank: MT921884, MT921916; MNHN-IM-2019-1711; • 1 spm; ibid; GenBank: MT921885, MT921917; MNHN-IM-2019-1712; • 1 spm; ibid; GenBank: MT921886, MT921918; MNHN-IM-2019-1713; • 1 spm; ibid; GenBank: MT921887, MT921919; MNHN-IM-2019-1714.

BELIZE • 4 spms; Carrie Bow Cay, NE Cay, Glover’s Reef; 15 Aug. 1973; CCRE - Caribbean Coral Reef Ecosystems, Belize; RS Houbrick leg.; intertidal on rocks; USNM 1620286 (ex USNM 771083).

BRITISH VIRGIN ISLANDS • 5 spms; Tortola; Kjaer leg.; USNM 6488; • 4 spms; Jost Van Dyke Island, Little Harbour; Apr. 1958; Smithsonian - Bredin Caribbean Expedition; R/V Freelance; shore coll.; USNM 683404.

COLOMBIA • 4 spms; New Grenada, Sabanilla; USNM 103165; • 11 spms; “Albatross”, Sabanilla; USNM 193614; • 7 spms; New Grenada, Sabanilla; USNM 224969.

CURAÇAO • 5 spms; Slangenbaai; 1 Dec. 1971; KB Meyer leg.; on rocks just above tide; USNM 702302; • 1 spm; Willemstad; 23 Jun. 1925; J Eldred leg.; tide pools just above high tide; USNM 889594; • 1 spm; Willemstad; 23 Jun. 1925; J Eldred leg.; tide pools just above high tide; USNM 889598; • 1 spm; S shore, beach in front of CARMABI research station; 12°07'20"N, 68°58'08"W; 18 May 2015; E Strong leg.; among cobbles; GenBank: MT921875, MT921907; USNM 1618949; • 1 spm; ibid; GenBank: MT921876, MT921908; USNM 1618950; • 1 spm; ibid; GenBank: MT921877, MT921909; USNM 1618951; • 1 spm; ibid; GenBank: MT921878, MT921910; USNM 1618952.

DOMINICA • 11 spms; Marigot; RG Fennah leg.; USNM 513253; • 3 spms; Scotts Head, windward coast; 27 Feb. 1966; Manning and Hobbs leg.; among rocks above or at high tide level; USNM 678753; • 1 spm; Prince Rupert Bayat Cabrits; 9 Mar. 1966; Hobbs and Manning leg.; rocky; USNM 678763; • 1 spm; W of Portsmouth; 19 Apr. 1939; Smithsonian - Bredin Caribbean Expedition; Nicholson, et al leg.; among boulders and off shingle beach; USNM 682469; • 81 spms; Berekua; 8 Feb. 1965; Smithsonian - Bredin Caribbean Expedition; WW Wirth leg.; on rocks on beach; USNM 709072.

DOMINICAN REPUBLIC • 1 spm; Oro Oro Beach [sic, possibly Playa Dorada], S. shore; [19°46'27"N, 70°38'30"W]; H Dodge leg.; USNM 1620275 (ex USNM 603900).

GRENADA • 5 spms; Saline Point; 14 Mar. 1956; Smithsonian - Bredin Caribbean Expedition; R/V Freelance; W Schmitt and F Chace leg.; USNM 714033.

GUADELOUPE • 46 spms; Aug. 1946; on rocks; USNM 1620270 (ex USNM 487939); • 1 spm; Plage de Malendure; 16°10'28"N, 61°46'47"W; 7 May 2012; KARUBENTHOS Expedition stn. GM7; GenBank: MT921870, MT921902; MNHN-IM-2009-26684; • 1 spm; Plage de Malendure; 16°10'28"N, 61°46'47"W; 1 Apr. 2017; D Lamy leg.; GenBank: MT921871, MT921903; MNHN-IM-2019-1703; • 1 spm; ibid; GenBank: MT921872, MT921904; MNHN-IM-2019-1704; • 1 spm; ibid; GenBank: MT921873, MT921905; MNHN-IM-2019-1705; • 1 spm; ibid; GenBank: MT921874, MT921906; MNHN-IM-2019-1706.

HAITI • 3 spms; Orcutt leg.; USNM 1620262 (ex USNM 383065); • 166 spms; Département de l’Ouest, Saltron; Orcutt leg.; USNM 1620267 (ex USNM 439970); • 6 spms; Département du Sud, Port-Salut; Orcutt leg.; USNM 1620268 (ex USNM 440004).

HONDURAS • 34 spms; Utilla Island; [16°05'47"N, 86°55'44"W]; Simpson leg.; USNM 1620264 (ex USNM 434879); • 1 spm; Webb leg.; USNM 1620266 (ex USNM 434885).

JAMAICA • 1 spm; St. Mary, Markham Hill; Orcutt leg.; USNM 377918; • 1 spm; Pt. Antonio; CWJ leg.; USNM 1620265 (ex USNM 434882); • 4 spms; Portland, near Buff Bay; Orcutt leg.; USNM 1620269 (ex USNM 441213); • 1 spm; Portland, Port Antonio; Vendeyes, Orcutt leg.; USNM 1620271 (ex USNM 518050); • 8 spms; St. Mary, 2 mi. N Port Maria; 18°24'19"N, 76°53'18"W; JI McCurkin leg.; 1 ft above splash line under cobbles; USNM 1620285 (ex USNM 770590).

MEXICO • 2 spms; Quintana Roo, Mujeres Island, Naval Station; 29 Mar. 1960; Smithsonian - Bredin Caribbean Expedition; R/V Blue Goose; W Schmitt leg.; USNM 662200; • 2 spms; Cozumel, 1 mi. N of San Miguel, in front of Cabanas del Caribe property; [20°31'59"N, 86°56'21"W]; 3 Apr. 1960; Smithsonian - Bredin Caribbean Expedition; R/V Blue Goose; H Rehder, W Schmitt and E Bousfield leg.; on rocks from above high tide to below water line; USNM 1620276 (ex USNM 662773); • 1 spm; Quintana Roo, Cozumel Island; 9 Apr. 1960; Smithsonian - Bredin Caribbean Expedition; R/V Blue Goose; H Rehder and E Bousfield leg.; splash pool high up; USNM 735218; • 1 spm; Quintana Roo, Ascension Bay, Suliman Pt. to 300 yds. to SW shore; 19 Apr. 1960; Smithsonian - Bredin Caribbean Expedition; R/V Blue Goose; W Schmitt, F Daiber, E Bousfield, J Clarke, H Rehder, Haynes and Harvey leg.; reef to sand flats; USNM 736379.

MONTSERRAT • 24 spms; Fox Bay, just N of Plymouth; 20 Apr. 1959; Smithsonian - Bredin Caribbean Expedition; R/V Caribee; black sand beach; USNM 1620281 (ex USNM 682493).

NETHERLANDS ANTILLES • 12 spms; St. Martin; Ford leg.; USNM 1620263 (ex USNM 434878).

PANAMA • 1 spm; San Blas; TG Thompson leg.; USNM 1620273 (ex USNM 597416); • 3 spms; Puerto Perme, NW of Cape Tiburon; [8°44'13"N, 77°32'41"W]; 14 Mar. 1953; RH Stewart leg.; USNM 1620279 (ex USNM 664216).

PUERTO RICO • 1 spm; Culebra, Ensenada Honda; USNM 161343; • 15 spms; E of Guanica, Tamarindo Beach; 23 Feb. 1958; GL Warmke leg.; under rocks; USNM 655599; • 2 spms; Fajardo; 10 Dec. 1951; J Weber leg.; USNM 1620277 (ex USNM 663091).

ST. LUCIA • 3 spms; Pigeon Island, N of Pigeon Island Club; 15 Apr. 1959; Smithsonian - Bredin Caribbean Expedition; R/V Caribee; sand and boulder flats around breakwater; USNM 682400.

ST. VINCENT AND THE GRENADINES • 9 spms; St. Vincent; Chamberlain leg.; USNM 1620261 (ex USNM 131804).

U.S. VIRGIN ISLANDS • 3 spms; St. Thomas; CB Adams leg.; USNM 1620259 (ex USNM 6419); • 1 spm; St. Croix; Helen F Dunn leg.; USNM 363994; • 9 spms; St. Croix, Davies Bay; Jan. 1936; HA Beatty leg.; from rocks; USNM 423961; • 12 spms; St. Thomas; Petit leg.; USNM 1620272 (ex USNM 530351); • 2 spms; St. Thomas, S end Magens Bay; M Jones leg.; rocks adjacent to beach; USNM 1620280 (ex USNM 666148); • 4 spms; St. John, Caneel Bay; [18°20'34"N, 64°47'13"W]; 25 Mar. 1958; Smithsonian - Bredin Caribbean Expedition; R/V Freelance; C Shuster and J Finlay leg.; USNM 1620284 (ex USNM 738775).

UNITED STATES • 2 spms; Miami Beach, South Beach, at jetty; [25°45'48"N, 80°07'45"W]; 12 Aug. 84; RS Houbrick leg.; intertidal rock and boulders; USNM 1620287 (ex USNM 842270); • 5 spms; Miami Beach, South Beach, at jetty; [25°45'48"N, 80°07'45"W]; 20 Nov. 83; RS Houbrick leg.; intertidal, on rocks and boulders; USNM 1620288 (ex USNM 842272); • 2 spms; South Miami Beach; 16 Jan. 1984; RS Houbrick leg.; USNM 1620289 (ex USNM 858456).

VENEZUELA • 5 spms; Cubagua Island; WL Schmidt leg.; low cliffs just back of sand beach; USNM 604065; • 1 spm; La Orchila Island; Sep. 1950; S Arias-Carbonell leg.; USNM 656012.

##### Description

. *Shell.* Shell (Figs 3C, 4H–O, 7A–L at left) medium-sized for the genus, solid, littoriniform, high-spired, consisting of 5+ (apex generally broken or corroded in adults) weakly convex whorls separated by impressed suture, last whorl occupying ca 75–80% of total shell height. Shell surface rarely smooth, usually covered with 15–17 even, incised spiral grooves extending over all shell height, especially well marked on base. Aperture ovoid, occupying ca 50% of total shell height, with or without sharp internal lirae, 7–11 in number, outer lip regularly convex, columellar pillar truncated above broad siphonal notch, callus adpressed to parietal and columellar areas, particularly expanded adapically, its outer edge thickened and slightly raised, parietal tooth strong, blunt. *Color* uniformly reddish brown to dark violet, parietal tooth and columellar callus often lighter. *Height* 11–13.5 mm.

*Radula*. Radula taenioglossate (Fig. 6A). Rachidian pentagonal, with broad basal plate, long basolateral extensions, and short, rounded, median basal projection (Fig. 6B, D). Upper lateral part of basal plate with robust, rounded basal denticle. Rachidian cutting edge rather narrow, comprising one-half width of tooth, but thick and heavily buttressed. Cutting edge sharply concave at midline, with narrowly rectangular, pointed median cusp, flanked by two to four smaller, robust, pointed denticles on each side. Lateral tooth with broad, high basal plate with central supporting ridge, rounded margins, and long lateral basal extension (Fig. 6C). Cutting edge broad with large, squarish, central cusp flanked by three to four inner and two to three sharp outer denticles. Marginal teeth elongate with curved paw-like tips (Fig. 6A, F). Inner marginal tooth with narrow flange along length of shaft outer edge, and with concave, rake-like tip with nine to ten rounded denticles. Outer marginal tooth with broad, membranous flange along distal outer edge, and with broad, bilobed tip bearing approximately 32 to 34 small, rounded denticles.

##### Distribution and ecology.

**Figure 8.**
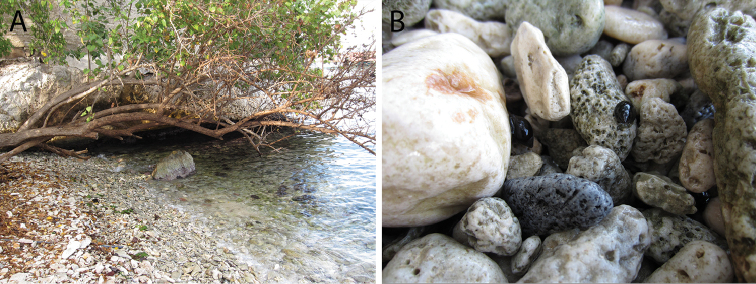
Habitat **A** Curaçao, S shore, beach in front of CARMABI research station **B***Supplanaxisnucleus* and *S.nancyae* individuals among cobbles.

The range of *Supplanaxisnancyae* extends from Miami Beach, Florida, in the north, to the northern coast of South America, from Veracruz, Mexico in the west (Tunnell 1974) and as far east as Trinidad and Tobago off Venezuela, including the Gulf of Mexico, Caribbean Sea and the Antillean Arc (Fig. 7). This species can occur in syntopy with *Supplanaxisnucleus* (Fig. 8) and museum lots across its range may comprise mixtures of the two species; ~ 30% of museum lots in the USNM with more than a single specimen included both species. Guadeloupe specimens of the two species are illustrated by Lamy and Pointier (2017: pl. 40, fig. 6A, B [*nucleus*], 6C–E [*nancyae*], both as *S.nucleus*). It is unknown whether this species also occurs in Bermuda.

##### Remarks.

Petit (2013: 9) questioned the precision of the type locality and presented circumstantial evidence that Petuch’s original material consisted of shells occupied by hermit crabs.

*Planaxisnancyae* was described based on two specimens, both unusually smooth and reddish for the species. In the absence of sequenced topotypic material, we are confident of the identity of the holotype based on our examination of more than 2,100 specimens of *Supplanaxis* from all over the Caribbean, and particularly material from Jamaica and the Virgin Islands that conforms with both our sequenced material from the Lesser Antilles and the Haiti holotype. The shell of *S.nancyae* differs from that of *S.nucleus* in its generally smaller size and lighter color, the regularly convex shape of the aperture, and its adapically expanded callus.

The protoconch figured by Houbrick (1987: fig. 17H) for *S.nucleus* may be that of *S.nancyae*, as all the adult shells in that lot (USNM 714035) represent the latter species. The radula of *S.nancyae* was described and figured as *S.nucleus* by Bandel (1984: fig. 59; pl. 2 figs 6, 8), as is evident from the morphology of the rachidian. Indeed, Bandel (1984) mistook the comparatively short lateral extensions of the rachidian described by Troschel (1858) as erroneous, but the latter were based on *S.nucleus* which has a broader cutting edge and comparatively shorter lateral extensions. The radular morphology of *S.nancyae* differs from that of *S.nucleus* primarily in its narrower and thicker cutting edge of the rachidian, the robustness and shape of the rachidian basal denticle, the narrower and more pointed rachidian central cusp, and the slightly greater number of denticles on the outer marginal tooth. There may be fewer flanking denticles on the rachidian in *S.nancyae* caused by fusion of the innermost denticles with the central cusp, as seen in the specimens examined herein from Guadeloupe (Fig. 6B, D), but the range of variation overlaps in the two species. These differences in tooth morphology strongly suggest the two species differ in trophic ecology, but this deserves further research. Regardless, in addition to features of the aperture of the shell, the characteristic rachidian provides diagnostic characters sufficient to separate *S.nancyae* from its co-occurring congener.

We note a strong resemblance with the fossil *Planaxisame* Woodring, 1928 (342, pl. 25, fig. 16), known from the Upper Pliocene of Jamaica (type locality) and from the Upper Miocene Cercado Formation of the Dominican Republic (Landau, pers. comm.). Woodring compared *Planaxisame* with *P.nucleus*, then the only known Recent Caribbean planaxid species, but the smooth forms of *Supplanaxisnancyae* are a better match, and we do not rule out that *P.ame* might turn out to be a senior synonym of *S.nancyae*.

#### Genus Hinea

Taxon classificationAnimaliaNeotaenioglossaPlanaxidae

Gray, 1847

81DD19EB-71C1-5D0C-B3CA-32925B1F9B04

##### Type species.

*Planaxismollis* G.B. Sowerby I, 1823 [= *Buccinumbrasilianum* Lamarck, 1822], by monotypy.

#### Hinea nucleola

Taxon classificationAnimaliaCerithioideaPlanaxidae

(Mörch, 1876) comb. nov.

3DA9787D-670D-540B-9B5F-E98DD2A679A4

Fig. 3E

Planaxis nucleola Mörch, 1876: 126

##### Holotype.

U.S. VIRGIN ISLANDS • 7.8 mm in height; St Croix; 1849; Ørsted leg.; ZMK 152749 (Fig. 3E).

##### Other material.

U.S. VIRGIN ISLANDS • 1 spm; St. John, Caneel Bay; USNM 1620829.

##### Description.

*Shell*. Shell small for the genus, solid, littoriniform, high-spired, consisting of 5+ (apex broken or corroded in the two known specimens) weakly convex whorls separated by impressed suture, last whorl occupying ca 80% of total shell height. Shell surface smooth, except incised spiral grooves, 0–2 adapically below suture and 2–8 on shell base. Aperture ovoid, occupying ca 40% of total shell height, with 0–4 low internal lirae, with distinct siphonal notch, narrow callus adpressed to parietal and columellar areas, parietal tooth strong, blunt. *Color* dark brown olive in holotype, to light orange brown with white parietal tooth and columellar callus. *Height* 7.8–8.4 mm.

##### Remarks.

*Planaxisnucleola* was described based on a single specimen, and the “probable holotype” (Fig. 3E) is in the Statens Naturhistoriske Museum [formerly Naturhistorisk Museum], Copenhagen. The type locality was St Croix, then in the Danish Virgin Islands [currently U.S. Virgin Islands].

*Planaxisnucleola* was explicitly separated from *P.nucleus* by Mörch (1876) by its much less acutely pointed shell and spiral grooves restricted to the base. It had never been illustrated, and we have not traced any citation of that species since Mörch (1876). It is currently indexed in MolluscaBase in the synonymy of *S.nucleus* based on Rosenberg (2009).

The genus-group name *Angiola* Dall, 1926, has long been in use for Caribbean planaxids (Houbrick 1987; Rosenberg 2009), but was synonymized with *Hinea* by Ponder (1988).

##### Discussion.

The sympatry and syntopy of *Supplanaxisnucleus* and *S.nancyae* raise the question of the evolutionary mechanism that might have led to the emergence of two species. Unexpectedly, the phylogenetic tree did not resolve *S.nucleus* and *S.nancyae* as sister taxa. A specimen of the Panamic *Supplanaxisplanicostatus* (G.B. Sowerby I, 1825) from Panama City [type locality: Galapagos Is; Fig. 3B], sequenced only for the 16S gene, is placed as sister to *S.nancyae*, albeit with no support. A further eastern Pacific *Supplanaxis* species is *S.obsoletus* (Menke, 1851) [type locality: Mazátlan, Pacific coast of Mexico]. The Caribbean and the Panamic species of *Supplanaxis* have obviously shared a long part of their evolutionary history, and more phylogenetic work needs to be done to properly assess their relationship.

## Supplementary Material

XML Treatment for Supplanaxis

XML Treatment for Supplanaxis nucleus

XML Treatment for Supplanaxis nancyae

XML Treatment for Hinea

XML Treatment for Hinea nucleola
